# Assessing aortic motion with automated 3D cine balanced steady state free precession cardiovascular magnetic resonance segmentation

**DOI:** 10.1016/j.jocmr.2024.101089

**Published:** 2024-08-30

**Authors:** Renske Merton, Daan Bosshardt, Gustav J. Strijkers, Aart J. Nederveen, Eric M. Schrauben, Pim van Ooij

**Affiliations:** a Amsterdam UMC location University of Amsterdam, Radiology and Nuclear Medicine, Meibergdreef 9, Amsterdam, the Netherlands; b Amsterdam Cardiovascular Sciences, Atherosclerosis and Ischemic Syndromes, Amsterdam, the Netherlands; c Amsterdam UMC location University of Amsterdam, Biomedical Engineering and Physics, Meibergdreef 9, Amsterdam, the Netherlands

**Keywords:** Aortic motion, 3D displacement, 3D cine bSSFP, Automated aortic segmentations

## Abstract

**Purpose:**

To apply a free-running three-dimensional (3D) cine balanced steady state free precession (bSSFP) cardiovascular magnetic resonance (CMR) framework in combination with artificial intelligence (AI) segmentations to quantify time-resolved aortic displacement, diameter and diameter change.

**Methods:**

In this prospective study, we implemented a free-running 3D cine bSSFP sequence with scan time of approximately 4 min facilitated by pseudo-spiral Cartesian undersampling and compressed-sensing reconstruction. Automated segmentation of the aorta in all cardiac timeframes was applied through the use of nnU-Net. Dynamic 3D motion maps were created for three repeated scans per volunteer, leading to the detailed quantification of aortic motion, as well as the measurement and change in diameter of the ascending aorta.

**Results:**

A total of 14 adult healthy volunteers (median age, 28 years (interquartile range [IQR]: 26.0–31.3), 6 females) were included. Automated segmentation compared to manual segmentation of the aorta test set showed a Dice score of 0.93 ± 0.02. The median (IQR) over all volunteers for the largest maximum and mean ascending aorta (AAo) displacement in the first scan was 13.0 (4.4) mm and 5.6 (2.4) mm, respectively. Peak mean diameter in the AAo was 25.9 (2.2) mm and peak mean diameter change was 1.4 (0.5) mm. The maximum individual variability over the three repeated scans of maximum and mean AAo displacement was 3.9 (1.6) mm and 2.2 (0.8) mm, respectively. The maximum individual variability of mean diameter and diameter change were 1.2 (0.5) mm and 0.9 (0.4) mm.

**Conclusion:**

A free-running 3D cine bSSFP CMR scan with a scan time of four minutes combined with an automated nnU-net segmentation consistently captured the aorta’s cardiac motion-related 4D displacement, diameter, and diameter change.

## Graphical Abstract

**Figure:**
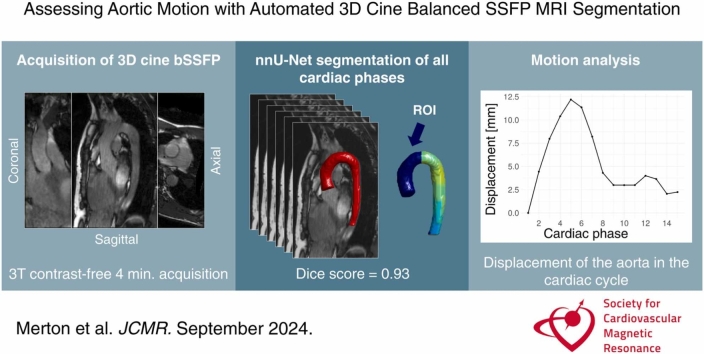

## 1 Introduction

Detailed information on aortic morphology plays an important role in the management of specific aortic abnormalities, such as bicuspid aortic valve and Marfan syndrome. Cardiovascular magnetic resonance (CMR) has provided vital insights in this realm, with its ability to quantify diameter, length, and cross-sections in both 2D and 3D imaging to understand changes between health and disease [1], [2], [3], [4]. However, the behavior of these morphological characteristics over the cyclical motion of the cardiac cycle has been under-investigated and is currently not used for diagnosis in clinical practice. Additionally, the motion of the aorta in the cardiac cycle is complex as it comprises both radial distension as a result of changing pressure and elongation and translation as a result of the mechanical contracting forces of the heart [5]. It is probable that significant interactions exist between microscopic tissue characteristics and the macroscopic dynamics of aortic behavior, where arterial stiffness plays a crucial role in restricting aortic motion. The characterization of this aortic motion can therefore be of great interest to gauge underlying biomechanics, relevant in aortic disease with increased risk of aortic dissection or rupture such as in Marfan syndrome, with the ultimate goal of treatment improvement.

Current research-driven aortic MR imaging techniques enable dynamic visualization and calculation of diameter and displacement in a single slice and orientation. However, they fall short in offering the ability to fully measure these parameters across the entire 3D thoracic aorta over time [6], [7], [8]. For example, assessment of distensibility often relies on a single plane through the mid-ascending aorta at the level of the pulmonary trunk and right pulmonary artery during breath-hold [8], [9], [10]. These measurements frequently neglect through-plane motion of the aorta which causes unrealistic changes in the 2D-derived aortic areas, while minor planning errors or unexpected breathing motion during acquisition can result in degraded scan quality rendering the final images unusable [8]. It has become evident that achieving high and isotropic resolution in 3D, along with time-resolved data, is vital for accurately determining the 3D displacement and diameter of the aorta.

Free-breathing 3D cine balanced steady state free-precession (bSSFP) CMR facilitates high and isotropic resolution time-resolved scans with coverage of the entire heart and/or thoracic aorta in a reasonable and clinically acceptable scan time [11], [12], [13]. With this scanning approach, measurements of aortic displacement and diameter have been confined to the differences between diastole and systole [11], due to the lengthy segmentation process required for segmenting entire 3D volumes in a bSSFP scan, excluding other cardiac phases. Concurrently, the development and implementation of (semi-)automated aortic segmentation techniques in CMR have demonstrated their practicality for both healthy and diseased states in 2D [8], [14], [15] and 3D [1], [16], [17], [18], [19], [20], [21], [22], [23]. Specifically, deep learning approaches using neural networks have proven its accuracy in segmenting 3D medical images [16], [17], [20], [21], [22], [23], [24], [25].

Leveraging these developments, the current study seeks to extend the diastolic-to-systolic method to a dynamic evaluation over all cardiac phases, made possible through the use of nnU-Net [26]. The aim is to provide detailed visualization and quantification of the heartbeat-related changes in aortic morphology over time. Therefore, a method for 4D analysis of displacement, diameter and diameter change of the thoracic aortic was developed from a single free-breathing cine bSSFP CMR scan with a duration of about 4 min without contrast enhancement. The heartbeat-related changes of these metrics were characterized in the ascending aorta (AAo) in healthy volunteers. Acquisition was repeated three times to evaluate the performance of the method.

## 2 Methods

### 2.1 Data acquisition

Fourteen healthy control subjects (self-reported absence of known cardiovascular disease; 6 women, 8 men ranging in age from 25–44 years (median age: 28 years (interquartile range [IQR]: 26.0–31.3))) underwent CMR on a 3 T scanner (Ingenia, Philips, Best, Netherlands) equipped with a dStream Anterior coil and in-house developed software modification for accelerated CMR called PROspective Undersampling in multiple Dimensions (PROUD) (https://mriresearch.amsterdam/software/aumcproudpatch/). The thoracic aorta was imaged using a free-running 3D cine bSSFP scan with retrospective cardiac binning and respiratory binning and correction [11]. Reconstructions were performed off-line in Matlab R2021a (The MathWorks Inc. Natick, Massachusetts) using ReconFrame (Gyrotools, Zurich, Switzerland) in combination with the Berkeley Advanced Reconstruction Toolbox [27] for non-linear compressed sensing reconstructions with a total variation sparsifying operator in the cardiac and respiratory dimension as previously described [11], [27]. This scan was repeated on the same day and two weeks later (referred to as test, retest, and rescan) to allow repeated measurements on the same volunteers, yielding a total of 42 datasets. Additional scan parameters were: isotropic spatial resolution (acquired and reconstructed) = (1.6 mm)^3^, field of view (FOV) = 256 × 256 × 88 mm^3^, Echo time/repetition time (TE/TR) = 1.7/3.4 ms, flip angle (FA) = 30°, minimum scan time = 3 min: 48 s and mean reconstructed temporal resolution = 67 ms (range: 50–84 ms) using 15 reconstructed cardiac phases. As a result of cardiac arrhythmia rejection some variability in scan time was introduced, with a mean scan time of 4 min: 00 s and 78% of scans with this duration or less. Three scans took longer than 4 min:15 s, with a maximum scanning time of 5 min: 29 s. Additionally, a standard clinical 2D cine bSSFP scan was planned orthogonal to the aortic arch between the brachiocephalic and left common carotid artery for diameter comparison. The arch was chosen to limit the effect of through-plane motion in order to provide comparable quantification. Scan parameters were: acquired / reconstructed resolution = 2.0 mm × 1.6 mm / 0.94 mm × 0.94 mm, slice thickness = 8 mm, FOV = 300 × 300 × 8 mm^3^, TE/TR = 1.47/2.95 ms, FA = 45°, temporal resolution = ∼50 ms (20 cardiac phases). The study was approved by the local medical ethics review committee. Informed consent was obtained from all subjects.

### 2.2 3D segmentation

A dataset of 84 manual segmentations of the aorta at end-systole and mid-diastole from sinotubular junction to the descending aorta below the level of the aortic root and above the liver

dome, excluding the subclavian and carotid arteries, was available from the 3D cine bSSFP scans of these 14 subjects. This allowed for the training of a nnU-net deep learning network [26]. The subjects were randomly divided into a nnU-net-training (10) and nnU-Net-test set (4) which corresponded to dataset sizes of (subjects x 2 phases x 3 scans) 60 vs. 24. The 3D full resolution version with five-fold cross-validation on the training data was used with 200 epochs. To avoid repeated data usage, a single subject (and their repeat scans) was never used in both training and validation. After training, this network was used to create segmentations for all cardiac phases of all scans.

#### 2.2.1 Motion analysis

Time-resolved displacement maps were derived after non-rigid registration of one end-diastolic reference segmentation (always the first timeframe) to all subsequent timeframes. Displacement was defined as the Euclidean distance between the reference and registered segmentation for each timeframe [11]. The non-rigid registration was based on iterative global deformation using $N_{c}$ Gaussian radial basis functions (G-RBF) with width *γ* [28]. For this study, 30 iterations were empirically determined with $N_{c}=10^{k1}$ and $\gamma =\frac{1}{{\left(2\times D_{\mathrm{mean}}\right)}^{k2}}$ where *k1* and *k2* linearly increase/decrease from 1 to 1.5/3 to 1.5 respectively and *D*_*mean*_ is the mean distance between the source and target meshes.

The 4D segmentations were also used to calculate time-resolved diameter maps by tracking the inward normal reaching the opposite wall per surface vertex, for each timeframe separately [29]. The diameter map of the end-diastolic reference was registered and subsequently interpolated to all subsequent timeframes and the difference in diameter between timeframes was defined as diameter change.

**Fig. 1:**
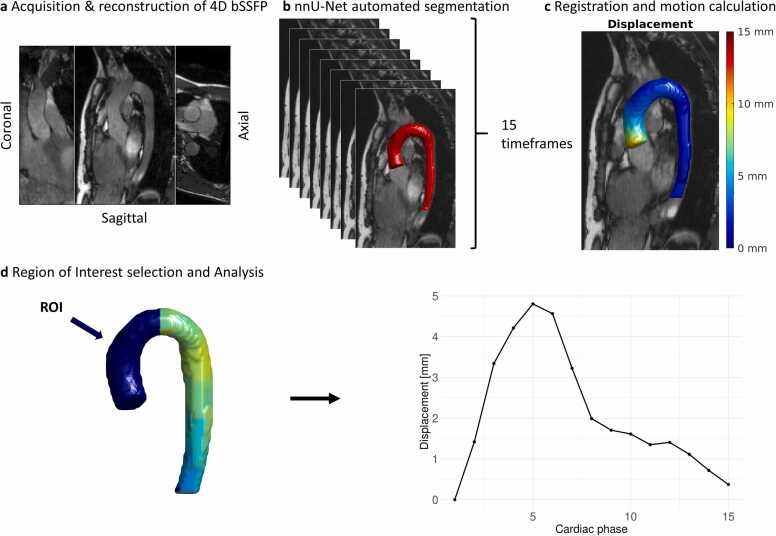
**a.** Acquisition of a 3D cine balanced steady state free precession (bSSFP) scan with compressed sensing reconstruction into a 4D dataset. **b.** Automated segmentation of fifteen cardiac phases using a trained nnU-net model. **c**) Registration of an end-diastolic reference phase (t = 1) to the other 14 phases and calculation of motion versus this reference frame. **d.** Selection of region of interest in the ascending aorta (AAo) and motion quantification of all cardiac phases. *ROI* region of interest,

The AAo region was defined as a bounding box from the sinotubular edge of the aortic segmentation up to the midpoint of the aortic arch on the reference phase and remained static for other phases. For assessing the temporal development of each metric, the mean value over the AAo vertices of each cardiac phase was plotted. This acquisition and analysis pipeline is schematically summarized in Fig. 1.

### 2.3 Statistics

All continuous variables except Dice score are expressed as median ( IQR) unless stated otherwise. To assess deep learning versus human segmentation performance, the nnU-Net-test set segmentations were compared using the Dice similarity coefficient (DSC). In a subset of ten volunteers a diameter measurement on the 2D cine bSSFP scan and the corresponding location on the 3D segmentation were compared using Pearson’s correlation and Bland-Altman analysis after a Shapiro-Wilk test was used to confirm the assumption of normality of the difference distribution. These diameters were measured in Materialise Mimics Medical version 24.0.0.427. Maximum variability between test-retest-rescan per volunteer was calculated as the largest measurement difference over all cardiac timeframes.

## 3 Results

**Fig. 2:**
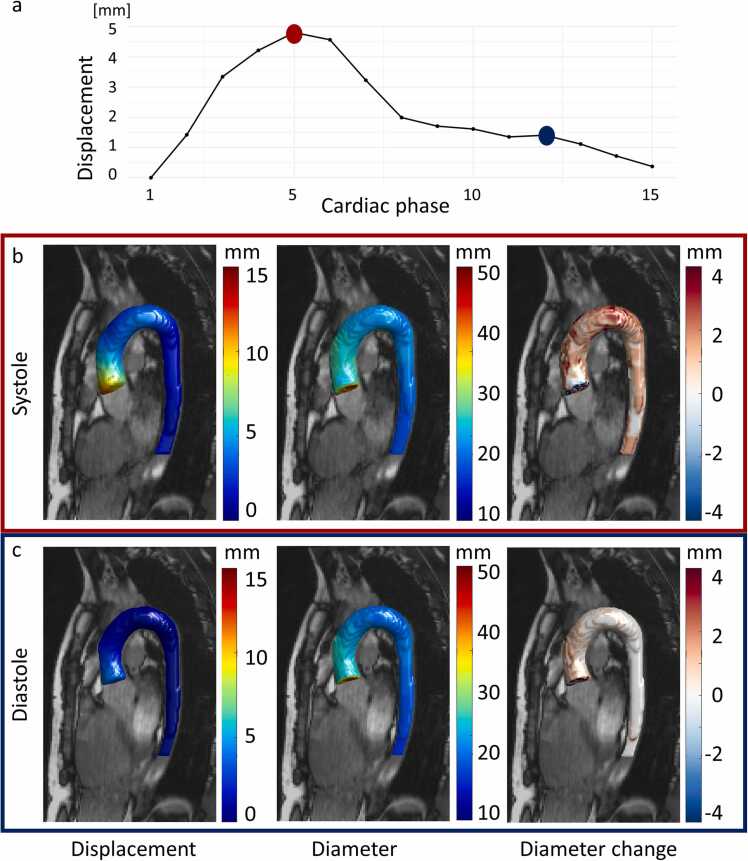
Aortic segmentations generated by nnU-Net for two cardiac phases with corresponding maps of displacement, diameter and diameter change for one example volunteer. **a.)** Curve of displacement averaged over ascending aorta, highlighting the systolic (red) and diastolic (blue) phases shown below. **b)** The systolic displacement map (left), diameter map (middle) and diameter change map (right) in mm overlaid on the corresponding 3D cine bSSFP. **c)** The same motion maps for the diastolic cardiac phase. *3D* three-dimensional, *bSSFP* balanced steady state free precession

One 3D cine bSSFP scan was excluded due to banding artefacts caused by inadequate shimming which led to poor image quality. Automated compared to manual segmentation of the nnU-Net-test set resulted in a Dice score of 0.93 ± 0.02. Dynamic motion maps were created to visualize the 3D aorta over the cardiac cycle portraying important morphology information such as displacement, diameter, and diameter change (Fig. 2 and Video 1).

**Fig. 3:**
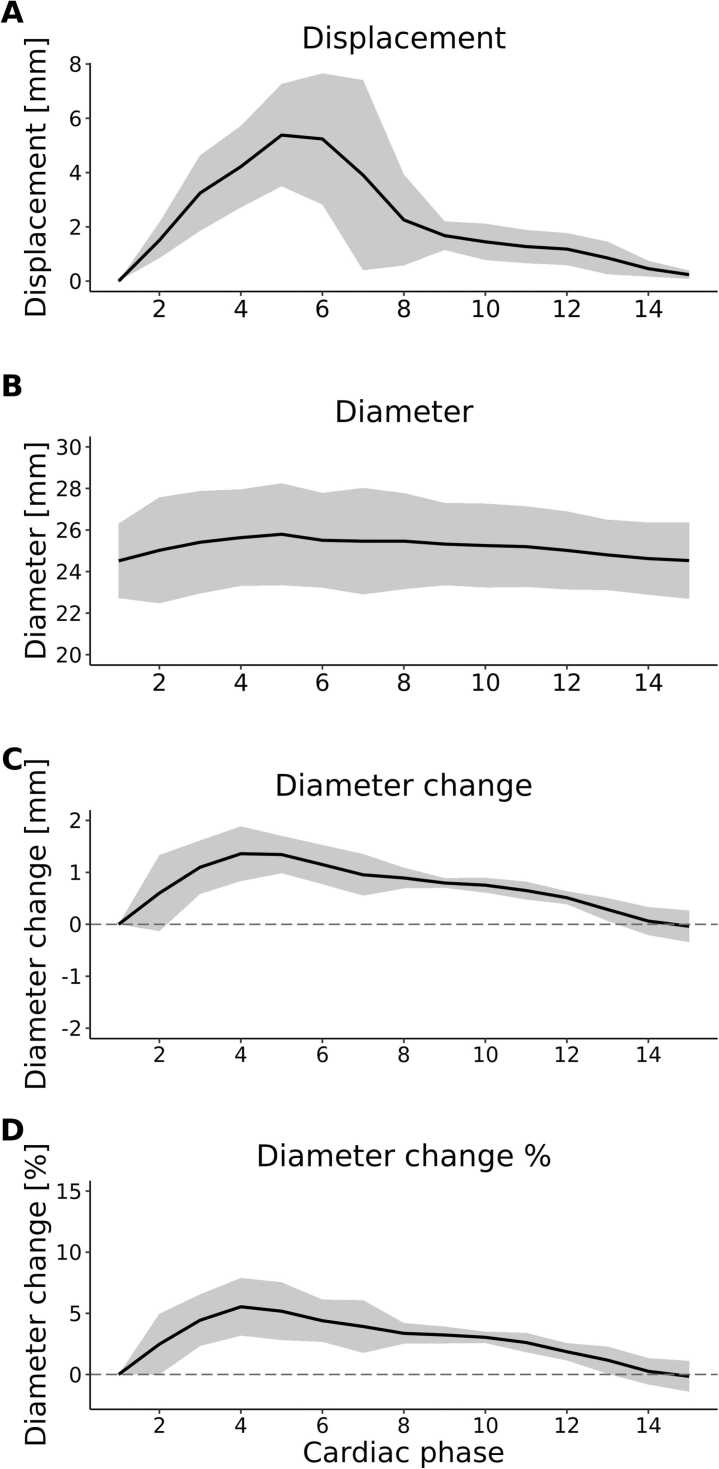
Graphs of a) dynamic displacement, b) diameter, c) diameter change and d) diameter change percentage with respect to the reference diameter averaged over the ascending aorta (median ± IQR) over all subjects. *IQR* interquartile range

Fig. 3 showcases the average displacement, diameter, and change in diameter (in mm and as percentage of the diameter at reference carciac phase one) across the AAo for the first scan of all subjects, presented as median (IQR). Across all volunteers, the largest maximum and average displacement of the AAo were 13.0 (4.4) mm and 5.6 (2.4) mm. Peak diameter averaged over the AAo was 25.9 (2.2) mm and peak diameter change was 1.4 (0.5) mm and 5.6 (2.1) %.

**Fig. 4:**
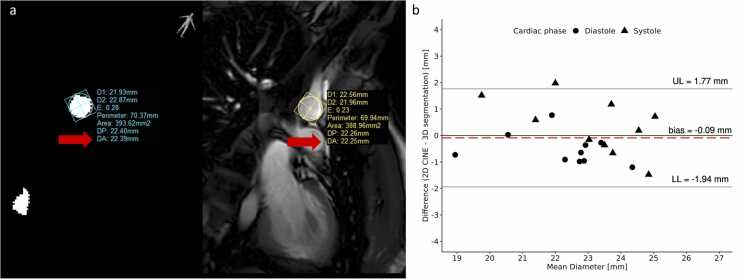
Example diameter analysis on the 2D cine and 3D automated segmentation. a) Representative case for diameter measurements on the 3D automated segmentation and 2D cine. Diameter based on area is used ($\mathrm{DA}=2*\mathrm{sqrt}(\frac{\mathrm{Area}}{\pi })$). b) Bland-Altman plot comparing the measurements on the 2D cine and 3D automated segmentation. *2D* two-dimensional, *3D* three-dimensional, *UL* upper limit, *LL* lower limit.

Fig. 4 shows the agreement between diameter measurements from the 2D cine and 3D segmentations. Across all volunteers, the systolic/diastolic diameter for 2D cine was 23.4 (1.3)/22.3 (0.7) mm and for 3D segmentations 23.4 (2.8)/23.1 (1.5). Pearson’s R between diameter as measured on 2D cine bSSFP and 3D segmentation was 0.85 and mean difference was −0.09 mm with LOA of ± 1.86 mm.

**Fig. 5:**
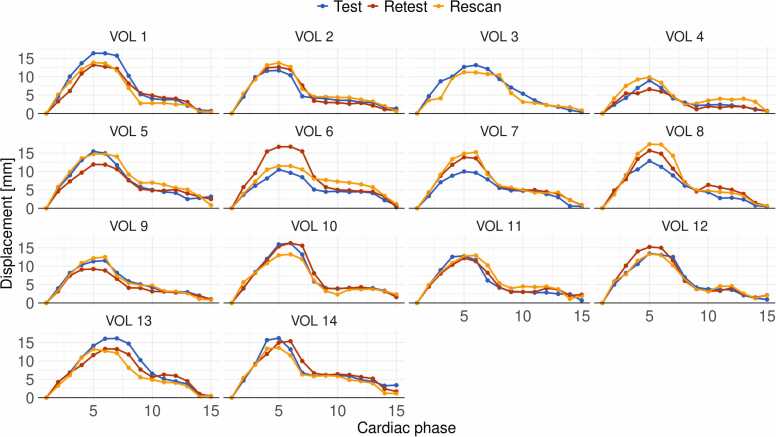
Maximum displacement over the AAo in mm of the test, retest, and rescan for all volunteers over all cardiac phases. *AAo* ascending aorta

Fig. 5 shows the test-retest-rescan comparisons of maximum AAo displacement over the cardiac cycle for all volunteers. The paired samples Wilcoxon test showed no difference in heartrate and systolic or diastolic pressures between the two measurement days (Table 1). The maximum individual variability over the three scans of maximum and average AAo displacement was 3.9 (1.6) mm and 2.2 (0.8) mm, respectively. The maximum individual variability of average AAo diameter and diameter change were 1.2 (0.5) mm and 0.9 (0.4) mm. The test-retest-rescan for average displacement, diameter, and diameter change can be found in supplemental Figs. S1-S3.

**Table 1:** Participant characteristics. Numbers are median (Q1 – Q3).

No. of participants	14	
Age (y)	28 (26 – 31)	
Female	6/14 (43%)	
Weight (kg)	75 (68 −81)	
Height (cm)	181 (174 – 188)	
BMI	22.8 (21.9 – 24.3)	
Scan session	1	2
Heart Rate (beats per minute)	60 (55 – 66)	59 (56 – 63)
Systolic Pressure (mmHg)	128 (122 – 131)	127 (121 – 132)
Diastolic Pressure (mmHg)	77 (74 – 81)	81 (77 – 82)

*BMI* body mass index.

## 4 Discussion

Over time, pathological dilation of the aortic wall may lead to alterations in the dynamic motion and distensibility of the aorta, highlighting the need for separate monitoring of these changes, as recommended by Burris et al. [30]. These morphological alterations will affect both the motion behavior and the inherent condition of the aortic wall. Here we introduce a novel combination of aortic CMR acquisition, reconstruction, and post processing to generate dynamic 3D motion maps which depict cyclical aortic motion including diameter and diameter change. The median curves from the first scan of all volunteers revealed a collective trend in these motion parameters for AAo. Variability over test/retest/rescan of AAo displacement was most pronounced during systole, with the majority of volunteers showing similar curves. In one case the automated segmentation failed due to loss of signal in the blood pool.

Beyond achieving strong segmentation agreement as indicated by the DSC, validating the quality of automated segmentation by comparing it with 2D cine diameter measurements revealed a mean difference nearly at 0 mm, with limits of agreement approximately within 2 mm. Considering that a single aorta specialist may obtain measurements that differ by 3 mm from identical images [31], this indicates a very favorable agreement with our automated method. When compared to 2D cine CMR, systolic diameter measurements using our method tend to exhibit a positive bias more frequently than diastolic measurements. This is likely due to the downward aortic motion as a result of cardiac contraction, combined with an 8 mm slice thickness leading to cross-section overestimation in 2D.

Over all volunteers, the largest maximum displacement was 13.0 (4.4) mm, whereas a previous study by these authors comparing one systolic and diastolic phase measured a mean maximum displacement of 11.0 mm [11]. Here the end-systolic phase was selected (typically heart phase 6) and then a diastolic phase 5 cardiac phases later (typically heart phase 11). The observed variation in displacement measurements underscores the value of this dynamic map, which provides measurements for each cardiac phase. The motion graphs give an impression of the motion ‘path’ of the aorta: a systolic peak followed by a diastolic plateau, which decreases at the end of diastole before the new cycle starts.

Given this context, direct comparison of measured values with literature values is challenging due to insufficiently detailed specification of the cardiac phase in the measured values. Kim et al. found a total displacement of the aortic root centerline-point of 7.34 ± 1.69 mm based on their CT-based method between a systolic and diastolic phase in a group of 13 patients without aneurysms (mean age of 48.6 ± 12.5 years) [25]. Plonek et al. retrospectively measured a longitudinal displacement of the aortic annulus of 11.6 ± 2.9 in a patient group of 73 patients referred for CMR (mean age of 45.2 ± 17.3) [7]. The maximum displacement values observed in our study were higher, at 13.0 (4.4) mm, and are believed to be the closest comparable metric. The elevated values observed may be attributable to the older age of the patient group compared to our volunteers, as age is associated with reduced aortic motion [7]. Alternatively, the differences could stem from measuring the displacement between peak and plateau phases rather than capturing the maximum displacement throughout the cardiac cycle.

The introduction of novel 4D methodologies presented in this paper brings to the forefront the issue of identifying relevant quantitative parameters. Despite the availability of 4D motion data, current studies continue to focus on reporting changes between end-systolic and diastolic phases. This is because comparing measurements necessitates boiling down the information to comprehensive figures [25]. However, as indicated, if the cardiac phases used for measurement are not specified in detail, a comparison of measures can be unreliable. One of the challenges in this field is facilitating a paradigm shift toward visualizing dynamic motion information and conducting meaningful analysis. This could include offering interactive tools that allow physicians to instantly select regions of interest and view corresponding motion graphs, enhancing investigative capabilities.

In future work it would be interesting to apply these methods to patients with Marfan syndrome to investigate whether this genetic disease presents with decreased aortic displacement and diameter change compared to healthy controls on MRI similar to CT [25] and how these measurements compare to other measures of arterial stiffness like pulse wave velocity from 4D flow CMR. In order to expand the use of the nnU-Net to groups of patients, it is expected that the nnU-Net-training set will have to be complemented with a set of manually segmented scans of the patient group to sustain segmentation performance.

## 5 Limitations

The generalizability of the evidence for reproducibility is limited by the population of relatively lean, healthy subjects that were used for these investigations. Nonetheless, it is expected that the presented method will generally translate to patients as long as there is no arrhythmia, which is detected by the scanner and leads to rejection of datapoints. For prolonged periods of very high variability this could result in scan abortion. In future research, it would be of interest to expand this cohort across a larger age and BMI range and to demonstrate its feasibility in patients. It is expected that the technique will perform well in the abovementioned Marfan patients, as they generally have low heart rate variability [32].

The current temporal resolution obtained with reconstruction to 15 cardiac phases resulted in smooth cyclic motion of the aorta and showed the feasibility of this technique. This temporal resolution might be a limitation when an increase in heart-rate variability is expected within patient populations. However, a strength of the presented acquisition is that it allows flexible reconstruction of temporal resolution, such that scan time does not change when the number of cardiac phases changes. Our preliminary investigations indicate that the current scan time provides sufficient data to reconstruct 30 cardiac phases. Future work should entail a thorough investigation of the optimal number of cardiac phases needed for assessment of these motion parameters.

Further, this study used a segmentation-based registration method for displacement calculations. While it is a benefit that this is not affected by signal variation in medical images as long as segmentations can be retrieved and it delivers a good estimate of whole-aorta motion, a downside is that registration/deformation is based purely on surface shape. Image based methods for registration are proposed in CT, such as b-spline image registration in vascular deformation mapping (VDM) [25], [30], [33], which potentially captures the local displacement more accurately in the presence of recognizable landmarks but must otherwise also make assumptions in the case of aorta elongation. However, it can be expected that the signal intensity variations in bSSFP CMR at 3 T make this approach more challenging for this modality. Another approach of note is the mechanics-informed snakes isogeometric analysis (MISIGA), where mid-surfaces are used as approximations of the aortic shapes. Here assumptions about wall-thickness and boundary conditions are used, which also have the limitation of not capturing local differences in wall-quality and related motion exactly as they actually occur [34].

The non-rigid registration in this method was based on iterative global deformation using $N_{c}$ G-RBFs that modelled the deformation of $N_{c}$ centers and is interpolated at all other source vertices, generating a smooth warp from source to target mesh. The number $N_{c}$ of centers impacts the computational efficiency and affects the registration. $N_{c}$ was determined empirically and the degree of freedom it represents underlines the abovementioned limitation that this registration is not landmark based.

One of the challenges encountered with bSSFP scans at 3 T involves the occurrence of artefacts stemming from stop-bands in the signal across the off-resonance spectrum, occasionally resulting in signal attenuation within the blood pool. Volunteer 3's retest experienced this issue, which currently stands as a limitation inherent in the selected protocol. Further addressing this challenge involves a reduction in the TR of the sequence alongside the adoption of a higher FA of 40 degrees, which promises to mitigate the influence of these dark bands and foster a more uniform signal behavior within the blood pool relative to off-resonance fluctuations. However, realization of this approach was hindered by specific absorption rate (SAR) restrictions; nevertheless, employing simpler radiofrequency (RF) pulses could potentially facilitate its implementation.

## 6 Conclusion

We have presented a method based on 3D cine bSSFP CMR combined with machine learning segmentation and non-rigid registration algorithms to visualize and quantify 4D aortic displacement and aortic diameter and diameter changes.

## CRediT authorship contribution statement

**Pim van Ooij:** Writing – review & editing, Supervision, Funding acquisition, Conceptualization. **Renske Merton:** Writing – original draft, Visualization, Software, Methodology, Formal analysis, Data curation, Conceptualization. **Daan Bosshardt:** Writing – review & editing. **Gustav J Strijkers:** Writing – review & editing, Supervision. **Aart J Nederveen:** Supervision. **Eric M Schrauben:** Writing – review & editing, Methodology, Conceptualization.

## Declaration of Competing Interest

The authors declare the following financial interests/personal relationships which may be considered as potential competing interests: Pim van Ooij serves as an editorial board member for JCMR. If there are other authors, they declare that they have no known competing financial interests or personal relationships that could have appeared to influence the work reported in this paper.

## Data Availability

For this study, we used the Amsterdam UMC ‘PROspective Undersampling in multiple Dimensions’ (PROUD) software patch (https://mriresearch.amsterdam/software/aumcproudpatch/). All data and software are available on reasonable request.

## Appendix A Supplementary material

.

**Video 1:** Aortic segmentation generated by nnU-Net for all cardiac phases with displacement map (left), diameter map (center), and diameter change map (right) in mm overlaid on the corresponding 3D cine for one example volunteer.

## Footnotes

Appendix A Supplementary data associated with this article can be found in the online version at doi:10.1016/j.jocmr.2024.101089.

## References

[1] Dietenbeck T. (2018). 3D aortic morphology and stiffness in MRI using semi-automated cylindrical active surface provides optimized description of the vascular effects of aging and hypertension. Comput Biol Med.

[2] Hiratzka LF, et al., *2010 ACCF/AHA/AATS/ACR/ASA/SCA/SCAI/SIR/STS/SVM guidelines for the diagnosis and management of patients with thoracic aortic disease: Executive summary: A report of the american college of cardiology foundation/american heart association task force on practice guidelines, american association for thoracic surgery, american college of radiology, american stroke association*, in Circulation. 2010, Lippincott Williams and Wilkins. 10.1002/ccd.22537 20687249

[3] Erbel R. (2014). in European Heart Journal.

[4] Ohyama Y. (2018). Advances in cardiovascular imaging imaging insights on the aorta in aging. Circ: Cardiovasc Imaging.

[5] Hoskins PR, Lawford PV, and Doyle BJ, *Cardiovascular Biomechanics*. Cardiovascular Biomechanics. 2017: Springer International Publishing. 1–462.

[6] Rengier F. (2012). Heartbeat-related distension and displacement of the thoracic aorta in healthy volunteers. Eur J Radiol.

[7] Plonek T. (2018). The evaluation of the aortic annulus displacement during cardiac cycle using magnetic resonance imaging. BMC Cardiovasc Disord.

[8] Cecelja M. (2022). Aortic distensibility measured by automated analysis of magnetic resonance imaging predicts adverse cardiovascular events in UK Biobank. J Am Heart Assoc.

[9] Redheuil A. (2014). Proximal aortic distensibility is an independent predictor of all-cause mortality and incident CV events. MESA Study.

[10] Shan Y., et al., *Comprehensive Assessment of Aortic Compliance and Brachial Endothelial Function Using 3.0-T High-Resolution MRI: A Feasibility Study*. 2012. 10.1097/RCT.0b013e31825b823e 22805674

[11] Merton R. (2024). Reproducibility of 3D thoracic aortic displacement from 3D cine balanced SSFP at 3 T without contrast enhancement. Magn Reson Med.

[12] Moghari M.H., van der Geest R.J., Brighenti M., Powell A.J. (2020). Cardiac magnetic resonance using fused 3D cine and 4D flow sequences:Validation of ventricular and blood flow measurements. Magn Reson Imaging.

[13] Feng L. (2018). Five-dimensional whole-heart sparse MRI. Magn Reson Med.

[14] Herment A. (2010). Automated segmentation of the aorta from phase contrast MR images: Validation against expert tracing in healthy volunteers and in patients with a dilated aorta. J Magn Reson Imaging.

[15] Odille F., Steeden J.A., Muthurangu V., Atkinson D. (2011). Automatic segmentation propagation of the aorta in real-time phase contrast MRI using nonrigid registration. J Magn Reson Imaging.

[16] Guo J. (2024). Deep learning‐based analysis of aortic morphology from three‐dimensional MRI. J Magn Reson Imaging.

[17] Sieren MM, et al., *Automated segmentation and quantification of the healthy and diseased aorta in CT angiographies using a dedicated deep learning approach*. 2021. 10.1007/s00330-021-08130-2 34170365

[18] Bustamante M. (2015). Atlas-based analysis of 4D flow CMR: Automated vessel segmentation and flow quantification. J Cardiovasc Magn Reson.

[19] Shahzad R. (2019). Quantification of aortic pulse wave velocity from a population based cohort: A fully automatic method. J Cardiovasc Magn Reson.

[20] Hepp T. (2020). Fully automated segmentation and shape analysis of the thoracic aorta in non-contrast-enhanced magnetic resonance images of the german national cohort study. J Thorac Imaging.

[21] Garrido-Oliver J. (2022). Machine learning for the automatic assessment of aortic rotational flow and wall shear stress from 4D flow cardiac magnetic resonance imaging. Eur Radiol.

[22] Berhane H. (2020). Fully automated 3D aortic segmentation of 4D flow MRI for hemodynamic analysis using deep learning. Magn Reson Med.

[23] Bustamante M. (2023). Automatic time-resolved cardiovascular segmentation of 4D flow MRI using deep learning. J Magn Reson Imaging.

[24] Chen C. (2020). Deep learning for cardiac image segmentation: a review. Front Cardiovasc Med.

[25] Kim T. (2023). Three-dimensional characterization of aortic root motion by vascular deformation mapping. J Clin Med.

[26] Isensee F. (2021). nnU-Net: a self-configuring method for deep learning-based biomedical image segmentation. Nat Methods.

[27] Uecker M. (2015). Berkeley advanced reconstruction toolbox. Proc Int Soc Mag Reson Med.

[28] Audenaert E.A. (2019). Cascaded statistical shape model based segmentation of the full lower limb in CT. Comput Methods Biomech Biomed Eng.

[29] van Ooij P, et al. *3D Linear Regression Analysis Reveals Relationships of 4D flow MRI-derived Aortic Dimensions with Age, Gender and Wall Shear Stress in Patients with Aortopathy*. in *Proc. Intl. Soc. Mag. Reson. 25.* 2017.

[30] Burris N.S., Hoff B.A., Kazerooni E.A., Ross B.D. (2017). Vascular Deformation Mapping (VDM) of thoracic aortic enlargement in aneurysmal disease and dissection. Tomogr (Ann Arbor, Mich ).

[31] Elefteriades J.A., Mukherjee S.K., Mojibian H. (2020). Discrepancies in measurement of the Thoracic Aorta: JACC review topic of the week. J Am Coll Cardiol.

[32] Cherkas A., Zhuraev R. (2016). A marked decrease in heart rate variability in marfan syndrome patients with confirmed FBN1 mutations. Cardiol J.

[33] Burris N.S. (2022). Vascular deformation mapping for CT surveillance of thoracic aortic aneurysm growth. Radiology.

[34] Cox A. (2022). Mechanics-informed snakes isogeometric analysis (MISIGA): an image-based method for the estimation of local deformation and strain in blood vessels. Eng Comput.

