# Supplementary material for: Assessing aortic motion with automated 3D cine balanced steady state free precession cardiovascular magnetic resonance segmentation
Source: J Cardiovasc Magn Reson. 2024 Aug 30;26(2):101089. doi: 10.1016/j.jocmr.2024.101089 (PMC11615597; doi:10.1016/j.jocmr.2024.101089)

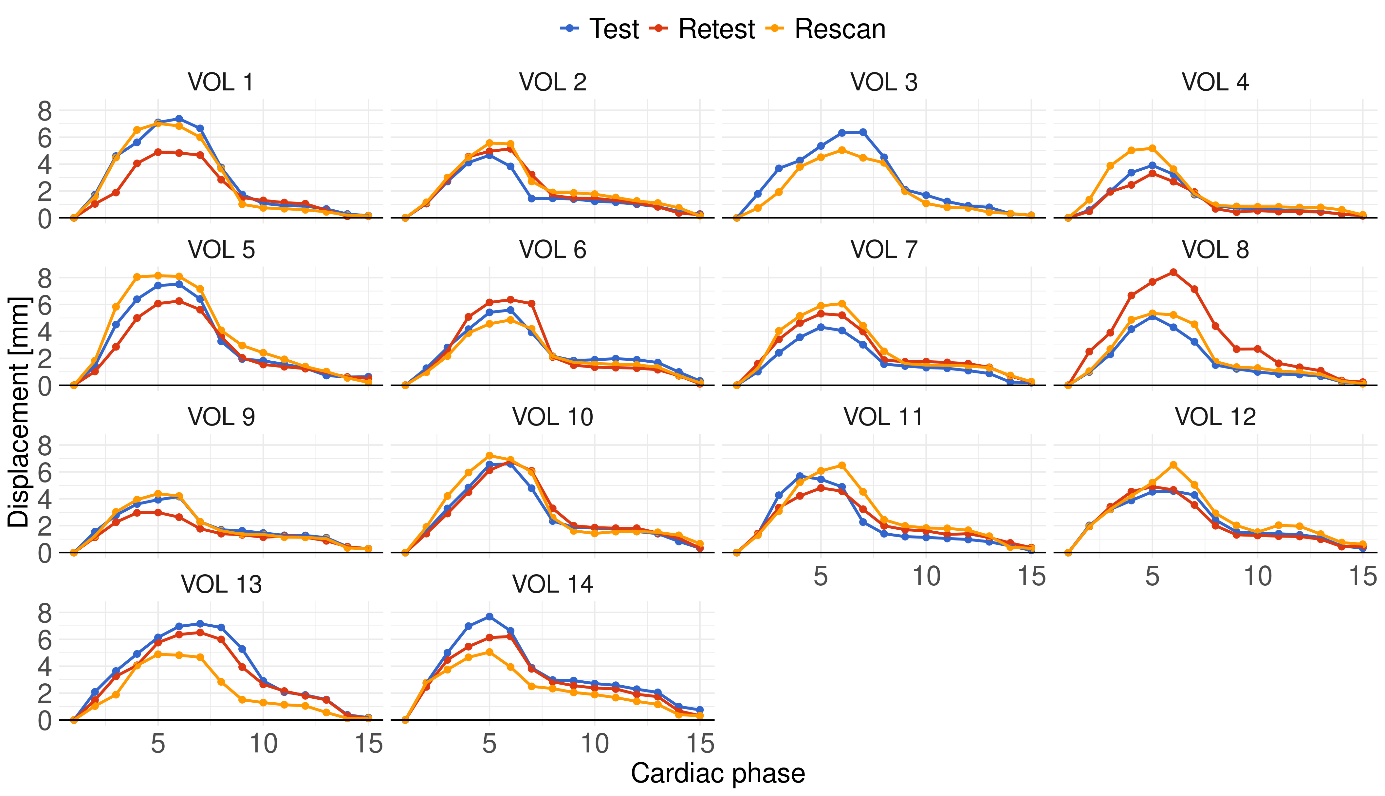
***Figure S1.*** *Average absolute displacement curves in mm relative to the reference phase (cardiac phase = 1) of the test, retest, and rescan of all volunteers over the subsequent cardiac phases.*


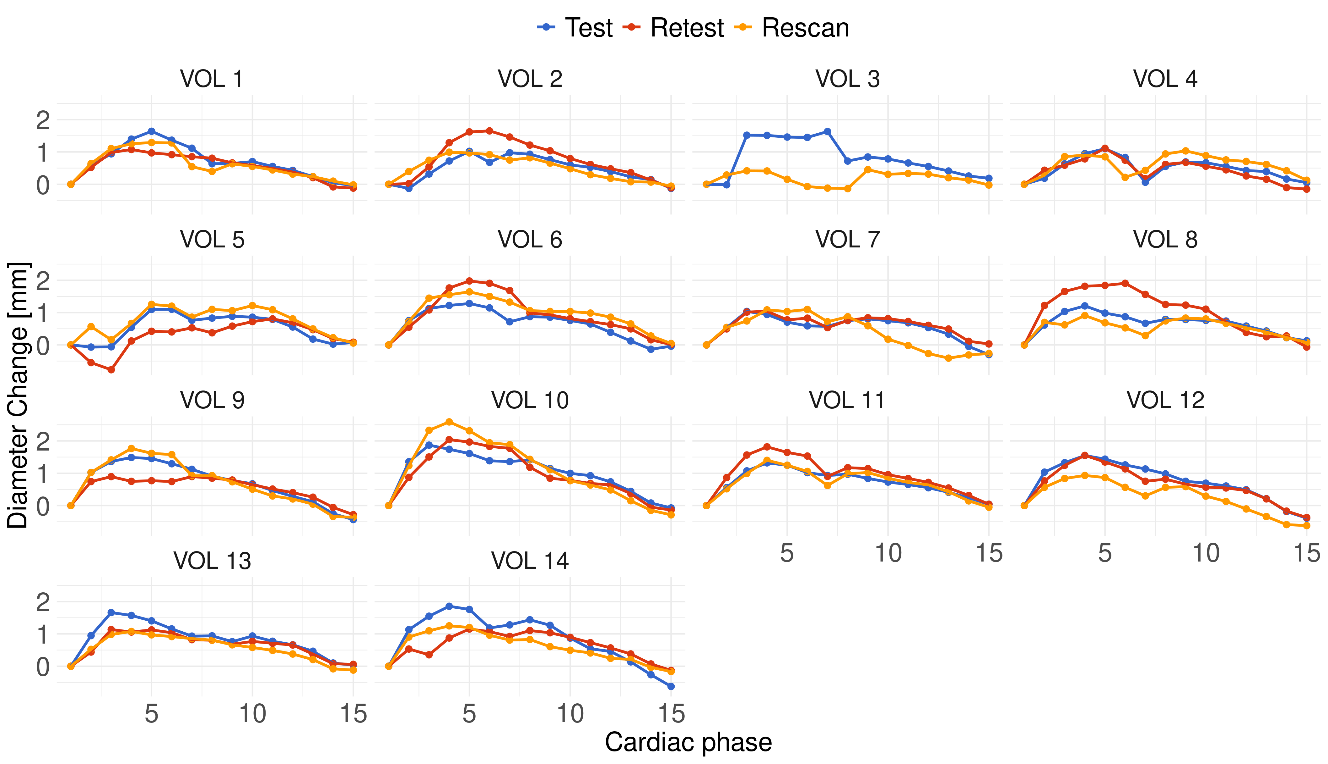


***Figure S2.*** *Mean absolute diameter change curves in mm relative to the reference phase (cardiac phase = 1) of the test, retest, and rescan of all volunteers over the subsequent cardiac phases.*

***Figure S3.*** *Mean absolute diameter curves in of the test, retest, and rescan of all volunteers over the subsequent cardiac phases.*


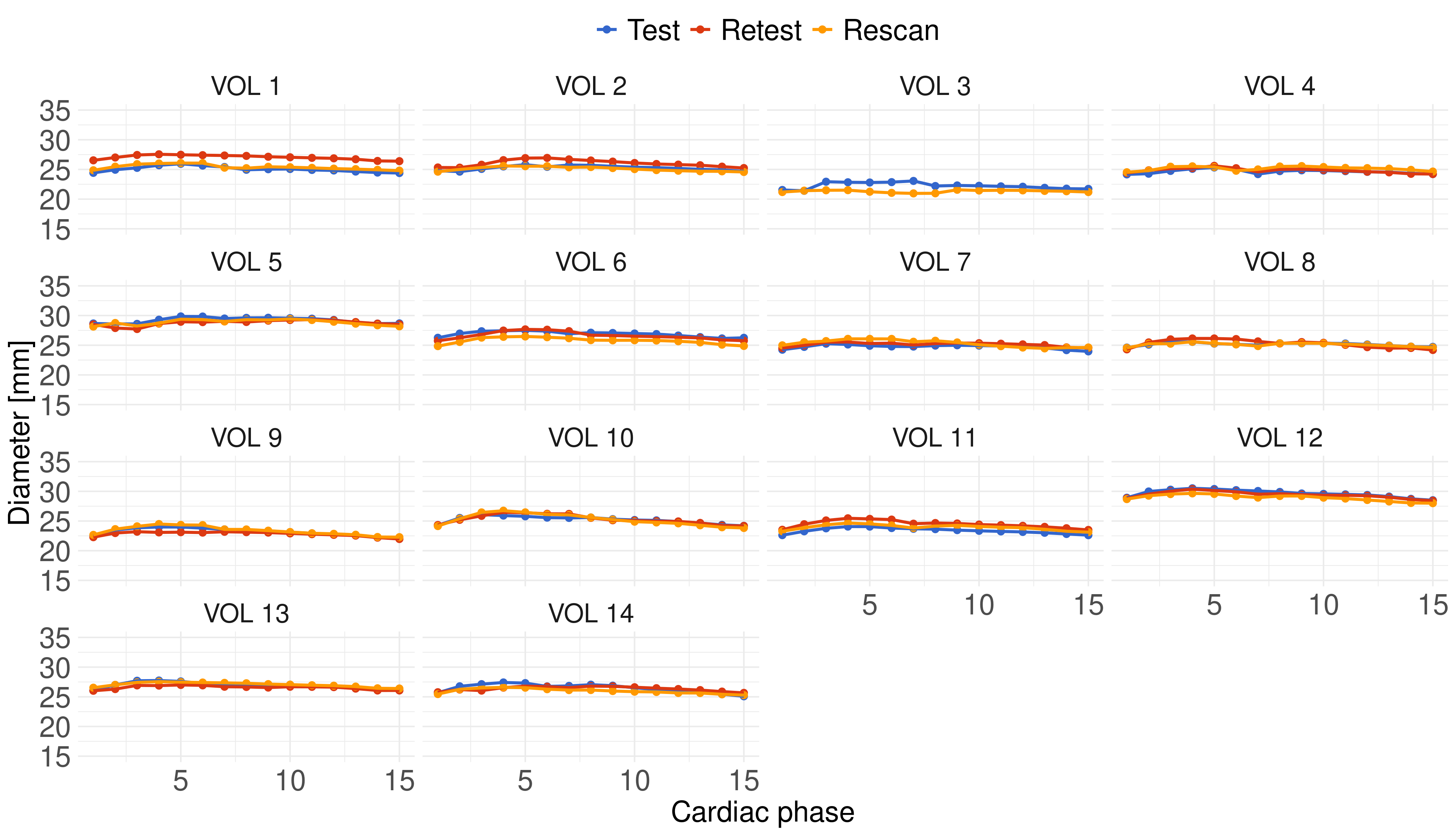

Supplement: Supplementary file 1 — Supplementary material [file mmc1.docx]
