# Supplementary figures and images for: Assessing aortic motion with automated 3D cine balanced steady state free precession cardiovascular magnetic resonance segmentation
Source: J Cardiovasc Magn Reson. 2024 Aug 30;26(2):101089. doi: 10.1016/j.jocmr.2024.101089 (PMC11615597; doi:10.1016/j.jocmr.2024.101089)

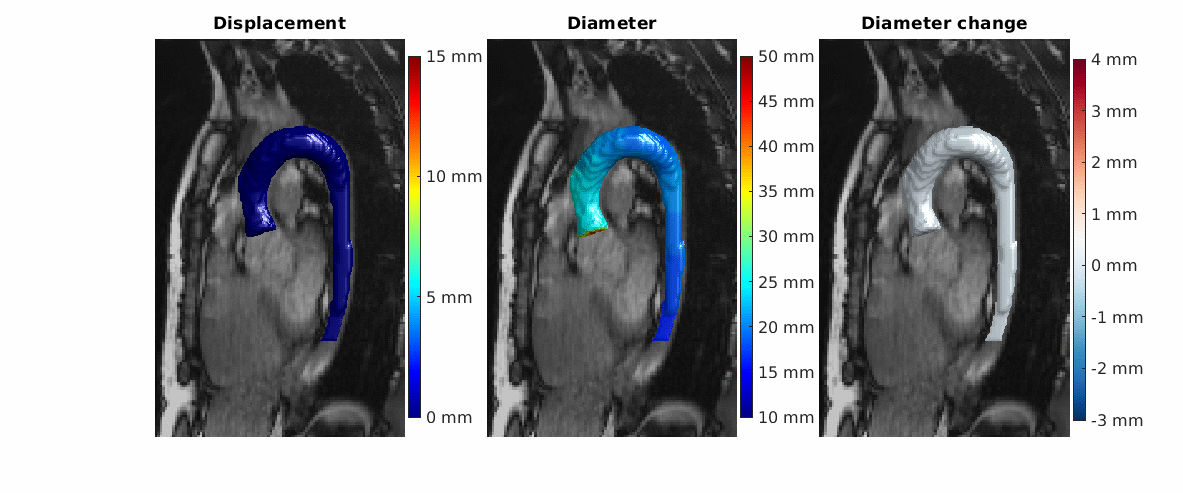

Supplement: Supplementary file 2 — Video 1: Aortic segmentation generated by nnU-Net for all cardiac phases with displacement map (left), diameter map (center), and diameter change map (right) in mm overlaid on the corresponding 3D cine for one example volunteer. [file mmc2.zip › Video1.GIF.gif]
